# Detection of Human polyomavirus 2 (HPyV2) in oyster samples in northern Brazil

**DOI:** 10.1186/s12985-020-01360-8

**Published:** 2020-06-26

**Authors:** Isabella Nogueira Abreu, Jacqueline Monteiro Cortinhas, Mike Barbosa dos Santos, Maria Alice Freitas Queiroz, Andréa Nazaré Monteiro Rangel da Silva, Izaura Maria Vieira Cayres-Vallinoto, Antonio Carlos Rosário Vallinoto

**Affiliations:** 1 Universidade Federal do Pará, Instituto de Ciências Biológicas, Laboratório de Virologia, Belém, Pará 66075-110 Brazil; 2 Instituto Evandro Chagas, Seção de Virologia, Ananindeua, Pará 67030-000 Brazil

**Keywords:** HPyV, Human polyomavirus 2, Oysters, Pará

## Abstract

**Background:**

Human polyomavirus 2 (HPyV2 or JCPyV) is persistent in the environment due to its excretion in urine and feces; it is detected in samples of wastewater, surface water and drinking water. A lack of basic sanitation and sewage collection results in the presence of this virus in food, especially in oysters, since they are bioaccumulators and are consumed in their natural form, thus posing a risk to human health.

**Methods:**

This study investigated the frequency of HPyV2 in samples of oysters marketed in northeastern Pará State, Brazil, and optimized a real-time PCR (qPCR) protocol for the detection of an endogenous oyster control. A total of 217 oysters in 22 pools from five municipalities in the state of Pará were analyzed. Samples underwent dissection and total maceration of oyster tissue using a viral concentration technique, followed by DNA extraction with phenol-chloroform and amplification of the VP1 region for molecular detection via qPCR.

**Results:**

HPyV2 was detected in 18.2% (4/22) of the pooled samples, with frequencies of 25, 20, 20 and 16% in the municipalities of Salinópolis, Augusto Corrêa, São Caetano de Odivelas and Curuçá, respectively. Notably, the sample pool from the municipality of Bragança did not have detectable HPyV2 and this was the only sampled location with a water treatment station. In this study, *Crassostrea* genus-specific primers (*AFL52* ribosomal RNA gene) of oyster were developed for use as an endogenous control in the qPCR analysis, which will be useful for future studies.

**Conclusions:**

The detection of HPyV2 in oyster samples commercialized in the state of Pará shows the circulation of this virus in the studied municipalities. Thus, it is necessary to implement measures for improving sewage collection and basic sanitation to avoid contamination of water and food with HPyV2.

## Background

Human polyomavirus 2 (HPyV2 or JCPyV) belongs to the *Polyomaviridae* family, genus *Betapolyomavirus*, and was one of the first human polyomaviruses to be isolated. Reactivation of this virus, in immunocompromised individuals, is associated with progressive multifocal leukoencephalopathy (PML), a neurological disease caused by progressive degeneration of the central nervous system (CNS) [1, 2].

HPyV2 infection is highly prevalent throughout the world and is characterized by a seropositivity of 70 to 80% [3]. The infection usually occurs in childhood and persists asymptomatically. In immunocompromised patients, viral reactivation may occur, leading to PML. Transmission can occur through the respiratory route, via horizontal transmission, through family cohabitation, fecal-oral and urine-oral routes and through renal transplantation [4–8]. One of the most common transmission routes is the urine-oral route because the virus establishes persistent infection in renal cells and is constantly excreted, which can result in water and food contamination [9, 10].

HPyV2 is detected in not only immunocompromised individuals but also healthy patients and environmental samples, such as seawater, treated water, treated sewage and mollusks, demonstrating that this virus is constantly excreted and is persistent in the environment. Due to its stability in the environment (HPyV2 remains stable for several months), and lack of seasonality, allowing use as a year-round indicator, this human-specific virus is considered a good marker of environmental contamination [11].

A large number of outbreaks caused by the consumption of contaminated oysters occur because oysters are bioaccumulator animals, which can filter out any microorganism present in water, such as viruses, bacteria and protozoa and store them in their tissues [12].

Studies investigating HPyV2 have focused on describing the prevalence of the virus, especially in transplanted patients, in individuals with immunosuppressive diseases and in environmental samples of water and sewage [7, 13, 14]. Studies involving the evaluation of environmental contamination in the context of HPyV2 detection in commercial oyster samples are extremely important because presence of this human-specific viral marker is highly indicative of contamination with human urine and feces.

Considering the increasing seafood production and consumption worldwide and, especially, in Brazil, the present study aims to identify HPyV2 contamination in oysters to prevent transmission of the virus, which can cause disease outbreaks in susceptible individuals. This study helps address the lack of data in this area and allows us to determine the potential risk that this virus represents in the development of diseases linked to the ingestion of contaminated mollusks, since these animals are ingested in their natural form.

## Materials and methods

The present study used environmental materials and did not involve humans. Thus, there was no need to submit the project to the ethics committee, according to resolution no. 466/2012 of the National Research Ethics Commission of Brazil (Comissão Nacional de Ética em Pesquisa - CONEP).

### Place of study

**Fig. 1:**
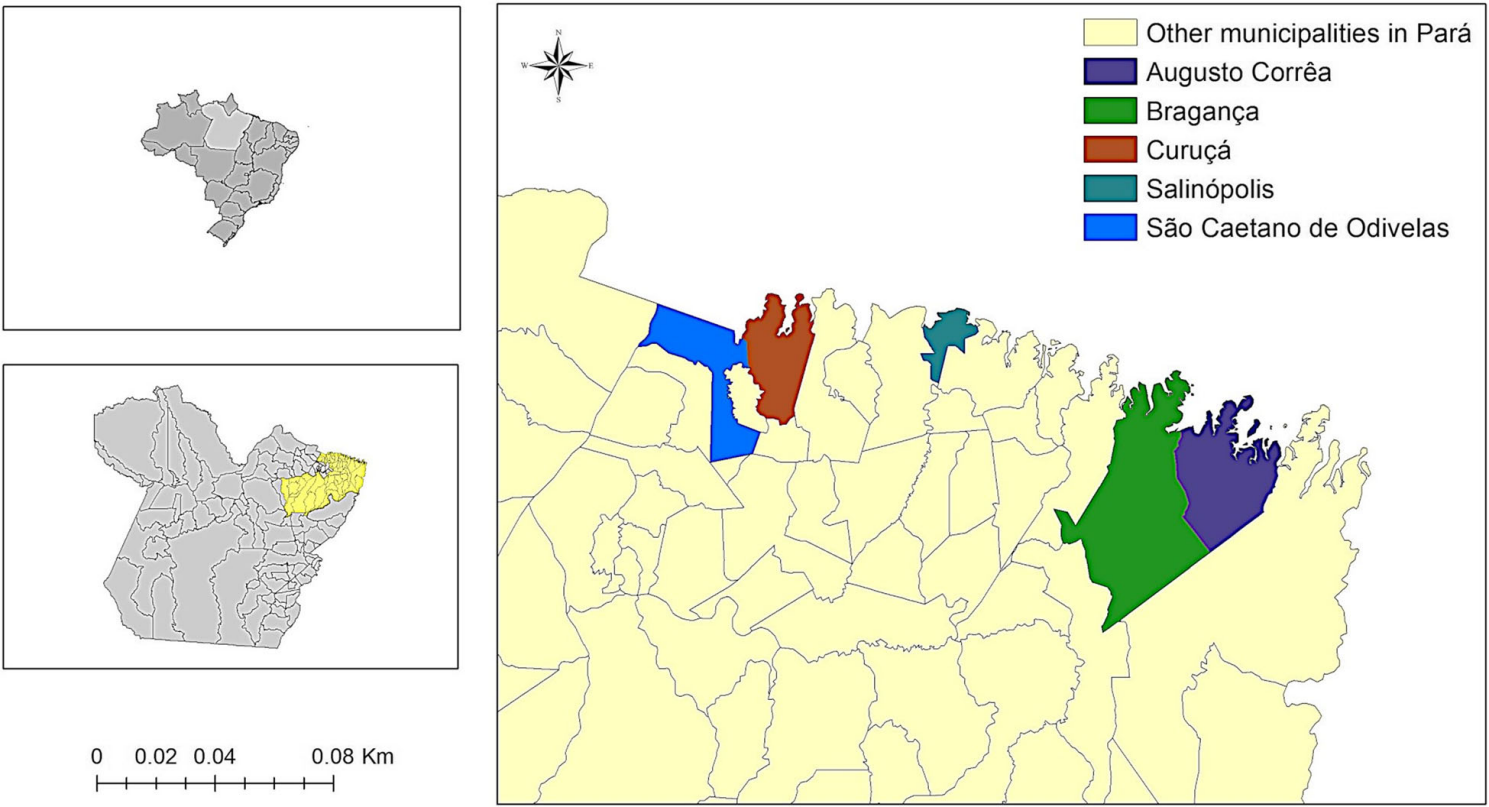
Map showing the location of the five municipalities where the oysters originated (Source: IBGE, 2019)

The state of Pará has 144 municipalities, with a territory that extends through an area of 1,245,759.305 km^2^ and an estimated population of 8,602,865 people [15]. The state is composed of six mesoregions and 22 microregions. Oysters were obtained commercially directly from growers in the Salgado and Bragantina microregions in the following municipalities (Fig. 1): Salinópolis, Curuçá and São Caetano de Odivelas (located in the Salgado region), Bragança and Augusto Corrêa (Bragantina region) [15].

### Sample collection and characterization

From June 2018 to September 2019, a total of 217 oysters were obtained commercially from growers in the microregions of Salgado and Bragantina, chosen as a result of the incentive for oyster production conducted by the Brazilian Service of Support to Micro and Small Enterprises (*Serviço Brasileiro de Apoio às Micro e Pequenas Empresas - SEBRAE*). From 23 to 52 oysters were obtained from each municipality. The oysters were completely dissected and macerated using an automatic homogenizer to obtain a liquid and homogeneous tissue solution. The oyster samples were tested using pools. One milliliter of each oyster’s macerate was collected to form 5- to 12-oyster pools. Each pool was considered a sample, totaling 22 samples (pools).

### Viral concentration method

From the pools formed the viral concentration method proposed by Rigotto et al. [16] and adapted from Lewis and Metcalf [17] was followed. From the homogenized oysters, 2 mL of tissue was added to 4 mL of the 10% tryptose phosphate broth (TPB) solution prepared in 0.05 M glycine buffer pH 9.0. Subsequently, the samples were homogenized in a shaker for 30 s and left under agitation at room temperature for 30 min.

After stirring, the solution was transferred to a clean tube, and an equal volume of chloroform-butanol was added (1:2 solution). Then, the solution was homogenized for 30 s in a shaker and centrifuged at 8000 rpm for 15 min at 4 °C. After centrifugation, the supernatant was transferred to a sterile tube, and an equal volume of polyethylene glycol (PEG) 6000 was added to a final concentration of 12%. The sample was then homogenized in a shaker for 30 s and left under constant agitation at 4 °C for 2 h. After this time, the samples were centrifuged at 8000 rpm for 20 min at 4 °C, and the concentrated material was solubilized in 3 mL of sterile MilliQ water. Next, the mixture was centrifuged again for 10 min at 8000 rpm at 4 °C, and the supernatant was collected. A 0.5 mL aliquot was separated, and the remainder was frozen at -80 °C.

Chloroform (150 mL, 30% of the sample volume) was added to a 0.5 mL aliquot. Then, the sample was agitated and centrifuged for 10 min at 8000 rpm at 4 °C. Subsequently the supernatant was transferred to new 1.5 mL tubes. The process was repeated, and finally, the samples were stored at -80 °C until use for DNA extraction.

### DNA extraction

Phenol-chloroform DNA extraction was adapted from Sambrook and Russell [18]. The protocol followed the steps of cell lysis, protein precipitation, DNA precipitation and hydration.

After extraction, the obtained DNA was quantified using a Qubit® 2.0 Fluorometer (Life Technologies, Carlsbad, California, USA) and reagents from a Qubit™ DNA Assay Kit (Life Technologies, USA) according to the protocol recommended by the manufacturer.

### Real-time PCR

Real-time PCR was performed using a TaqMan system to detect an 88-bp segment of the *JVGP5* gene of HPyV2, according to the method previously described by Pal et al. [19]. The sequences of the primers and probe used were as follows: Forward 5′-ATGTTTGCCAGTGATGATGAAAA-3, Reverse 5′-GGAAAGTCTTTAGGGTCTTCTACCTTT-3′ and probe FAM-5′-AGGATCCCAACACTCTACCCCACCTAAAAAGA-3′-MGB.

For qPCR protocol optimization, primers (Forward 5′-CCTCGATGTTGAATCAGGGATWA-3′ and Reverse 5′-TAGAAACCAACCTGGCTTACG-3′) and a probe (FAM-5′-ACCCTACGTGATTTGAGTTCAGACCG-3′-MGB) specific for amplification of 121 bp of the *AFL52* gene (large subunit ribosomal RNA) of oysters were designed using the online software Primer3 version 4.1.0. and were used as an endogenous control for the reaction (Figure supplementary 1). The chosen gene region was defined from the alignment of specific and conserved sequences in different oyster species (*Crassostrea gigas*, *C. gasar*, *and C. angulata)* based on the NCBI database.

Samples were tested in singleplex with specific and endogenous gene primers and probes (Thermo Fisher, Carlsbad, California, USA) using the Applied Biosystems Step One Plus Real-Time PCR device (Thermo Fisher, Carlsbad, California, USA). In each reaction, 1X MasterMix, H_2_O, 10 μM primers, 5 μM probe and 50 ng DNA were used and were subjected to the following cycling conditions: 10 min at 95 °C and then 40 cycles of 15 s at 95 °C and 1 min at 60 °C.

### Data analysis

The frequency of infection by HPyV2 was determined by direct counting (number of positive samples/total sample per municipality × 100).

## Results

Of the 22 pools (217 tissue samples) analyzed using the qPCR technique, 4 (18.2%) showed amplification of the HPyV2 genome (Table 1). All municipalities had samples positive for HPyV2, except for the municipality of Bragança, which did not have detectable HPyV2 genomic DNA present.

**Table 1:** Prevalence of qPCR positive according to the municipalities

**qPCR results**	**Salinópolis** n (%)	**Bragança** n (%)	**Curuçá** n (%)	**Augusto Correa** n (%)	**São Caetano de Odivelas** n (%)	**TOTAL** n (%)
Positive	1 (25)	–	1 (16)	1 (20)	1 (20)	18.2
Negative	4 (75)	2 (100)	6 (84)	5 (80)	5 (80)	81.8

In analysis of the frequency distribution of HPyV2 in the five municipalities studied, four were positive for HPyV2 (Table 1). The highest frequency was found in the municipality of Salinópolis, with 25% of the samples positive, followed by Augusto Corrêa (20%) and São Caetano de Odivelas (16%) in the municipality of Curuçá.

Table 2 shows the number of oysters obtained from each municipality, the number of pools analyzed and the number of positive samples in each location.

**Table 2:** Number of oysters obtained from each municipality and the number of pools analyzed

**Municipality**	**Number of oysters**	**Pools**	**HPyV2 positive pools**
Augusto Corrêa	50	5	1
Bragança	23	2	0
Curuçá	51	6	1
Salinópolis	41	4	1
São Caetano de Odivelas	52	5	1
**Total**	**217**	**22**	**4**

From the analysis, the amplification of endogenous control was observed in all samples (Figure supplementary 2). Threshold cycle (TC) values ranged from 18 to 32 between endogenous control samples, but it was 36 to the HPyV2 positive sample.

## Discussion

The present study is the first to identify HPyV2 contamination in oysters marketed in northern Brazil, especially in the northeastern region of Pará. Investigation of viruses in food is extremely important and has become a requirement in the field of public health, although there is no systematic inspection or even legislation that establishes criteria for food safety related to the presence of viruses in food [20].

In Brazil, there are bodies responsible for assessing water quality and food but that aim to analyze only the presence of bacteria. The detection of HPyV2 found in this study reinforces the need for inclusion of not only microbiological but also viral analyses, taking into account the low level of basic sanitation in the state of Pará [21].

In the present study, the presence of HPyV2 was identified in 18.2% of the samples analyzed, indicating circulation of the virus in the human population of the investigated municipalities, which may result in possible contamination via water intake, water consumption, or consumption of bivalve mollusks.

To date, the presence of HPyV2 has been detected in oyster samples in only two studies. In Barcelona, Spain, Bofill-Mas et al. [22] identified HPyV2 in 5/10 samples (six samples of oysters and four samples of mussels), while in Florianopolis, Souza et al. [23] observed the presence of HPyV2 in only 1/33 samples of oysters from suppliers. Therefore, at the national level, the detection rate observed in the present study is higher, given that 18.2% (4/22) of the samples were positive. It is worth to note that Florianopolis, the capital of Santa Catarina State, located in southern Brazil, has better water treatment stations and socioeconomic factors, as compared to the Pará State, thus reflecting the prevalence differences observed between the studies.

In the State of Pará, studies were performed demonstrating viral and microbiological contamination in culture water and oysters; however, these studies focused only on investigations of bacteria and viruses responsible for causing gastroenteritis because they have a greater impact on the population, which presents characteristic symptoms [12, 24, 25].

According to data from the National Sanitation Information System (Sistema Nacional de Informações sobre Saneamento - SNIS), referring to the data from 2017, only the municipality of Bragança has a Water Treatment Station (WTS), which may explain the absence of positive samples in this municipality. In other municipalities, water treatment is performed by simple disinfection, which favors water contamination. No data regarding the municipality of Curuçá were recorded [21].

Among the municipalities studied, Salinópolis had the highest contamination by HPyV2, which can be explained by the abovementioned simple sewage disinfection, the large-scale movement of tourists and swimmers who frequent this region and the presence of several shacks close to the shore that do not have adequate basic sanitation. The difference in total samples varied mainly in the municipality of Bragança, in which it was not possible to obtain 50 units of oysters due to difficulty in contacting the cultivator. Therefore the difference in HPyV2 genome detection for this municipality may potentially be due to undersampling.

HPyV2 is present in high concentrations in the environment, up to 10^3^ GC/g [26], and is used as a marker of environmental contamination due to its persistence in wastewater and surface water in several countries, the high stability of the virus in the environment and its specificity to the human host [27].

Several studies have demonstrated HPyV2 detection in different sample types, its excretion in urine and its detection in water and sewage samples, suggesting that there is a failure in sewage collection and water treatment, which makes it necessary to create laws to evaluate viral contamination in water and food samples and to determine an appropriate destination for sewage collection and treatment to avoid contamination of the water supply and rivers.

The search for viruses in environmental samples requires optimization of a qPCR protocol and development of specific primers as endogenous controls because several inhibitors can interfere with the reaction. The optimization for the endogenous control established in this study is pioneering because primers with specific sequences from a conserved ribosomal region were used for tree species of oysters that are grown in Brazil and in other countries, namely, *Crassostrea gigas*, *C. gasar*, *and C. angulata*.

In recent years, the increased production and consequent consumption of oysters in Pará was favored by the establishment of specific projects for education and training of oyster farmers in the Salgado and Bragantina microregions [28] but has not been accompanied by improvements in basic sanitation. The present study is of great importance as a public health alert for the northeastern region of the state of Pará, where HPyV2 was observed in commercial oysters, mainly in local restaurants and in the capital of Belém. Oysters are considered an aphrodisiac food rich in proteins and nutrients and are eaten raw, steamed, sautéed or grilled, making the consumption of this seafood quite common. Thus, the need to create laws to evaluate viral contamination in water and in seafood often consumed by the population and for improvements in sewage collection and basic sanitation is evident.

The results presented here highlight the need for further studies investigating HPyV2 contamination in the human population and in the water sources of the studied municipalities as the presence of this human-specific viral marker is highly indicative of poor water quality, especially when we consider that this virus can be linked with human colorectal cancer [29–31] or even undergo reactivation in immunosuppressed individuals, who have a high risk of developing PML [32].

## Conclusion

The detection of HPyV2 in oysters draws attention to the need to assess HPyV2 in this type of food because current laws only require the monitoring of bacterial contamination, which does not ensure complete quality and safety for those who consume oysters in their natural form. Additionally, The present study showed that an optimized protocol may be effective for investigating and detecting HPyV2 in oyster samples, which may be useful in future studies and in microbiological control of this seafood.

## Supplementary information

**Additional file 1: Fig. S1.** Nucleotide sequence alignment of oyster (*Crassotera* spp.) *AFL52* rRNA (Amplicon 121 bp).

**Additional file 2: Fig. S2.** Plot showing amplification of the endogenous oyster control (in blue) and positive samples for human Polyomavirus 2 (in red) determined via real-time PCR.

## Data Availability

The datasets in this study are available from the corresponding author on reasonable request.

## Abbreviations

HPyV2: Human polyomavirus 2
qPCR: Real-time PCR
PML: Progressive Multifocal Leukoencephalopathy
CNS: Central nervous system
TPB: Tryptose phosphate broth
PEG: Polyethylene glycol
TC: Threshold Cycle
WTS: Water Treatment Station
NCBI: National Center for Biotechnology Information
