# Supplementary figures and images for: Detection of Human polyomavirus 2 (HPyV2) in oyster samples in northern Brazil
Source: Virol J. 2020 Jun 26;17:85. doi: 10.1186/s12985-020-01360-8 (PMC7318511; doi:10.1186/s12985-020-01360-8)

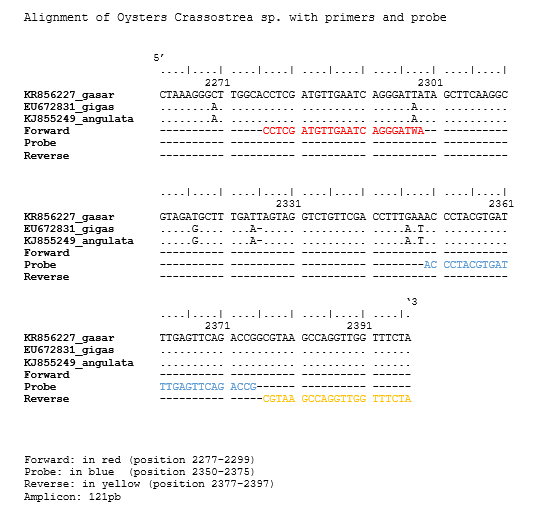

Supplement: Supplementary file 1 — Additional file 1: Fig. S1. Nucleotide sequence alignment of oyster (Crassotera spp.) AFL52 rRNA (Amplicon 121 bp). [file 12985_2020_1360_MOESM1_ESM.tiff]

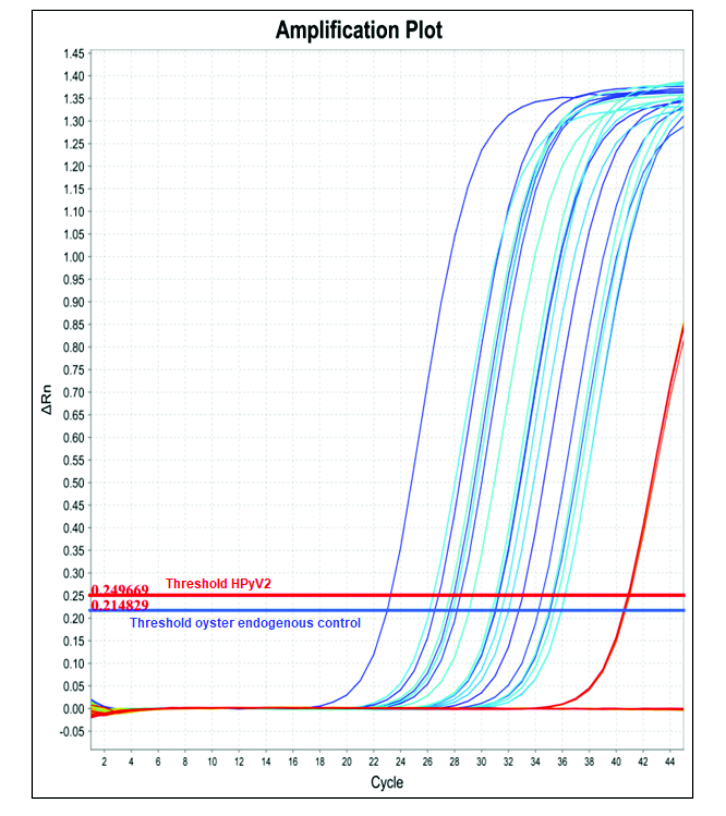

Supplement: Supplementary file 2 — Additional file 2: Fig. S2. Plot showing amplification of the endogenous oyster control (in blue) and positive samples for human Polyomavirus 2 (in red) determined via real-time PCR. [file 12985_2020_1360_MOESM2_ESM.tiff]
